# Evaluation of the fermentation of high gravity thick sugar beet juice worts for efficient bioethanol production

**DOI:** 10.1186/1754-6834-6-158

**Published:** 2013-11-08

**Authors:** Piotr Dziugan, Maria Balcerek, Katarzyna Pielech-Przybylska, Piotr Patelski

**Affiliations:** 1 Department of Fermentation Technology, Institute of Fermentation Technology and Microbiology, Lodz University of Technology, 90-924 Wolczanska, Lodz 171/173, Poland; 2 Department of Spirit and Yeast Technology, Institute of Fermentation Technology and Microbiology, Lodz University of Technology, 90-924 Wolczanska, Lodz 171/173, Poland

**Keywords:** Bioethanol, Fermentation, Thick juice, Sugar beet, High gravity wort, Yeast

## Abstract

**Background:**

Sugar beet and intermediates of sugar beet processing are considered to be very attractive feedstock for ethanol production due to their content of fermentable sugars. In particular, the processing of the intermediates into ethanol is considerably facilitated because it does not require pretreatment or enzymatic treatment in contrast to production from starch raw materials. Moreover, the advantage of thick juice is high solid substance and saccharose content which eliminates problems with the storability of this feedstock.

**Results:**

The objective of this study were to investigate bioethanol production from thick juice worts and the effects of their concentration, the type of mineral supplement, as well as the dose of yeast inoculum on fermentation dynamics and ethanol yield.

The obtained results show that to ensure efficient ethanolic fermentation of high gravity thick juice worts, one needs to use a yeast strain with high ethanol tolerance and a large amount of inoculum. The highest ethanol yield (94.9 ± 2.8% of the theoretical yield) and sugars intake of 96.5 ± 2.9% were obtained after the fermentation of wort with an extract content of 250 g/kg supplemented with diammonium hydrogen phosphate (0.3 g/L of wort) and inoculated with 2 g of Ethanol Red dry yeast per L of wort. An increase in extract content in the fermentation medium from 250 g/L to 280 g/kg resulted in decreased efficiency of the process. Also the distillates originating from worts with an extract content of 250 g/kg were characterized by lower acetaldehyde concentration than those obtained from worts with an extract content of 280 g/kg.

**Conclusions:**

Under the favorable conditions determined in our experiments, 38.9 ± 1.2 L of 100% (v/v) ethyl alcohol can be produced from 100 kg of thick juice. The obtained results show that the selection of process conditions and the yeast for the fermentation of worts with a higher sugar content can improve the economic performance of the alcohol-distilling industry due to more efficient ethanol production, reduced consumption of cooling water, and energy for ethanol distillation, as well as a decreased volume of fermentation stillage.

## Background

Biofuels are defined as solid (biochar), liquid (bioethanol, biobutanol, biodiesel) and gaseous (biogas, biosyngas, biohydrogen) fuels that are mainly derived from biomass. Traditionally, sugar substrates derived from food crops such as sugar cane, corn (maize) and sugar beet have been the preferred feedstock for the production of biofuels [1].

Bioethanol can be produced from all feedstock that contain mono-, oligo- and polysaccharides (for example, starch and cellulose) [2]. An advantage of raw materials containing simple sugars and disaccharides, such as saccharose, is the simplified technology of extraction to the water medium, followed by fermentation to ethanol without the need of using additional technological operations connected with chemical or enzymatic hydrolysis, which could significantly increase the costs of biosynthesis [2]. From an economic point of view and in comparison with cereals, sugar beet and beet-processing intermediates containing saccharose are very good raw materials for ethanol production due to their content of fermentable sugars (saccharose) [3,4].

The production of ethanol from sugar beet-processing intermediates (raw, thin, and thick juices) and from byproducts (molasses) constitutes an alternative solution for sugar factories interested in a combined production of sugar and bioethanol. Furthermore, the use of intermediate products of sugar beet processing as raw materials for bioethanol production could be attractive for distilleries located near the sugar factories, as it would minimize high transportation costs. Cooperation between these factories could lead to increased production and utilization of the capacity of both types of facilities.

Very high gravity (VHG) processes are extremely attractive and promising for bioethanol production as they allow significant improvements in overall productivity, thus minimizing production costs thanks to energy savings [5]. On the other hand, the use of VHG technology imposes greater stress on yeast cells, which has been associated with the loss of yeast viability during VHG fermentation, a reduced rate, and incomplete fermentation [6]. Thus, the successful implementation of VHG technology in bioethanol production requires the use of yeast strains that can efficiently ferment high sugar concentrations (>250 g/L) [7]. Such strains must be resistant to the multiple stresses found in the process, including the osmotic stress that results from high sugar concentration, the ethanol stress at the end of fermentation, the anaerobic conditions established in large-scale bioreactors, and the cell recycling procedures for the utilization of the yeast biomass for several consecutive fermentation cycles [8,9].

Balcerek *at al*. [10] investigated the effect of various strains of the yeast *Saccharomyces cerevisiae* (*S. cerevisiae*) on the dynamics and efficiency of alcoholic fermentation of thick juice worts. The authors tested strains designated as M1, M2, M3 (from the Pure Culture Collection of the Institute of Fermentation Technology and Microbiology, Lodz University of Technology), commonly used for the fermentation of molasses worts, as well as strains designated as Bc-16, D-2, As-4 (purchased from the yeast factory in Maszewo Lęborskie, Poland), used for the fermentation of mashes based on starch raw materials. It was found that *S. cerevisiae* strains M1 and M2 dynamically and efficiently (89 to 94% of the theoretical yield) fermented thick juice worts with an extract concentration of 200 g/kg and 250 g/kg, whereas the strain D-2 preferred less dense worts (extract concentration of 200 g/kg). Gumienna *et al.*[4] evaluated the efficiency of alcoholic fermentation of sugar beet and its processing intermediates using commercial yeast strains such as Ethanol Red^®^ and Fermiol (Fermentis Division S.I. Lesaffre, France). Balcerek and Pielech [11] also tested the Ethanol Red^®^ yeast strain for the fermentation of triticale starch mashes with a solid substance concentration of approximately 23%. The obtained results showed high ethanol yields (87.54 ± 0.46% to 88.30 ± 0.46% of the theoretical yield).

According to the declaration of the producer (Fermentis Division S.I.), Ethanol Red^®^ is a specially selected strain that was developed for the ethanol industry. With a high ethanol tolerance, this fast acting strain displays higher alcohol yields and maintains higher cell viability, especially during VHG fermentation. Ethanol Red^®^ is particularly well-suited for sugar substrates (sweet juices, molasses) and also saccharified mashes [12].

The objective of the presented study was to determine the effect of thick juice worts concentration, the type of mineral supplements, and the dose of yeast inoculum on the dynamics and efficiency of alcoholic fermentation.

## Results and discussion

### Chemical characteristics of thick juice

The chemical composition of thick juice applied in this study was typical of sugar beet processing intermediates (see Table 1). The high content of saccharose (598.4 g/kg) is advantageous from the technological point of view because it promotes a high yield of ethanol from the raw material. Our results are consistent with the data reported by Ranković *et al.*[13] with one exception related to the total nitrogen content. The thick juice described by Ranković *et al.*[13] contained four times less total nitrogen (1.4 g/kg) than the raw material used in our study (5.6 g/kg). The differences in the content of nitrogen compounds are probably related to the sugar beet varieties processed in sugar factories in Poland and Serbia [14], and to different sugar beet cultivation conditions and the technology used for processing it into thick juice (Table 1).

**Table 1:** Chemical composition of raw material

**Physicochemical parameters**	**Thick juice**
Solid substance (g/kg)	685.2 ± 11.5
pH	7.4 ± 0.2
Reducing sugars as invert sugar (g/kg)	3.1 ± 0.4
Saccharose (g/kg)	598.4 ± 12.5
Total nitrogen (g/kg)	5.6 ± 0.4
Volatile acids as acetic acid (g/kg)	4.4 ± 0.2

Results expressed as mean values ± standard error (n = 3).

The chemical composition of the investigated thick juice makes this intermediate product of sugar beet processing an attractive feedstock for alcoholic fermentation. Thick juice is only subjected to initial dilution, pH adjustment, and supplementation with inexpensive mineral sources of nitrogen for the yeast (if needed). This makes the overall process of bioethanol production from thick sugar beet juice relatively simple in comparison to production from starch-based raw materials, which require the liberation of starch (by pressure cooking or by thorough grinding) and its liquefaction and saccharification [11].

### The effect of process conditions on fermentation dynamics and the results of fermentation of high gravity thick juice worts

**Figure 1:**
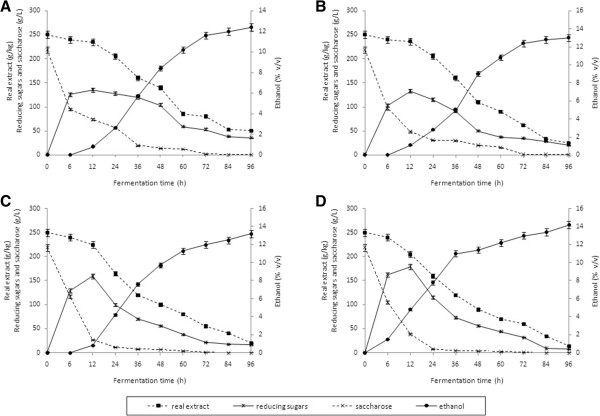
**Fermentation dynamics of thick juice worts with an extract content of 250 g/kg. (A)** Inoculum content of 1.0 g/L; (NH_4_)_2_HPO_4_. **(B)** Inoculum content of 1.0 g/L; (NH_4_)_2_HPO_4_ + MgSO_4_ · 7 H_2_O. **(C)** Inoculum content of 1.5 g/L; (NH_4_)_2_HPO_4_. **(D)** Inoculum content of 2.0 g/L; (NH_4_)_2_HPO_4_. MgSO_4_ ·7 H_2_O.

**Figure 2:**
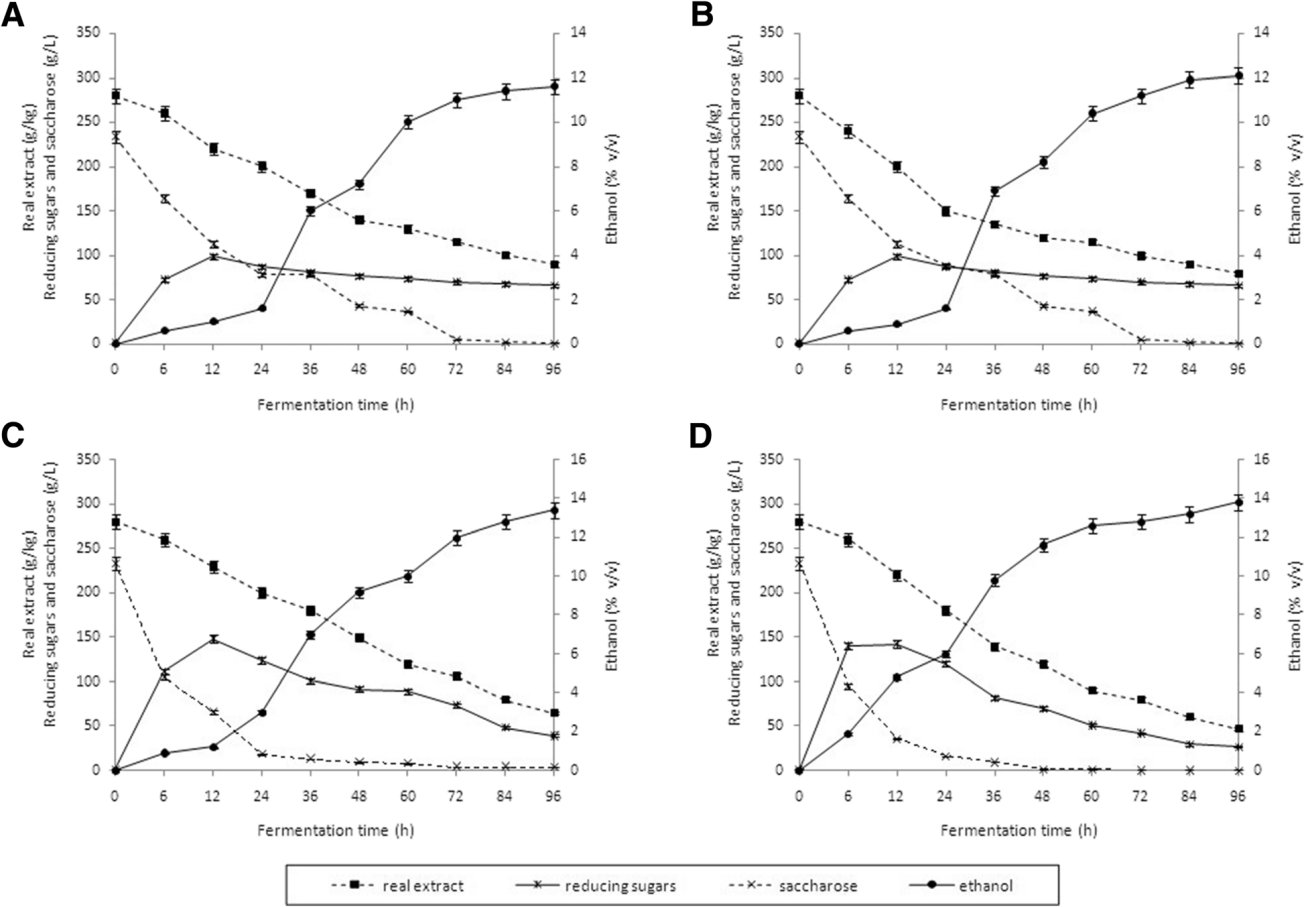
**Fermentation dynamics of thick juice worts with an extract of 280 g/kg. (A)** Inoculum content of 1.0 g/L; (NH_4_)_2_HPO_4_. **(B)** I noculum content of 1.0 g/L; (NH_4_)_2_HPO_4_ + MgSO_4_ · 7 H_2_O. **(C)** I noculum content of 1.5 g/L; (NH_4_)_2_HPO_4_. **(D)** Inoculum content of 2.0 g/L; (NH_4_)_2_HPO_4_.

**Figure 3:**
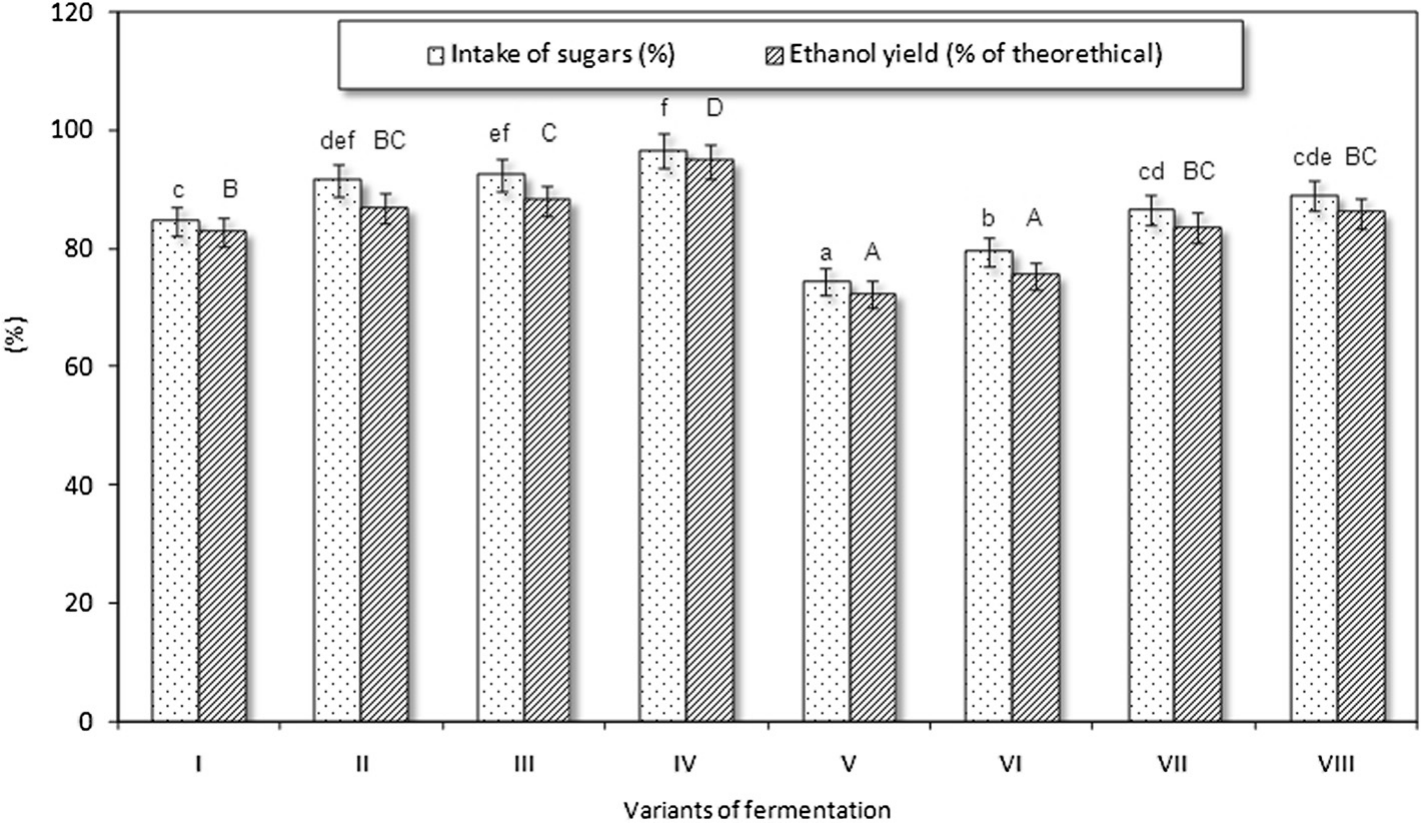
**Fermentation results of thick juice worts.** Different letters indicate significant differences (*P* <0.05) between the means of intake of sugars (lowercase letters) and ethanol yield (capital letters). Variants of fermentation: I, extract content of 250 g/kg, inoculum content of 1.0 g/L; (NH_4_)_2_HPO_4_. II, extract content of 250 g/kg, inoculum content of 1.0 g/L; (NH_4_)_2_HPO_4_ + MgSO_4_ · 7 H_2_O. III, extract content of 250 g/kg, inoculum content of 1.5 g/L; (NH_4_)_2_HPO_4_. IV, extract content of 250 g/kg; inoculum content of 2.0 g/L; (NH_4_)_2_HPO_4_. V, extract content of 280 g/kg, inoculum content of 1.0 g/L; (NH_4_)_2_HPO_4_. VI, extract content of 280 g/kg, inoculum content of 1.0 g/L; (NH_4_)_2_HPO_4_ + MgSO_4_ · 7 H_2_O. VII, extract content of 280 g/kg, inoculum content of 1.5 g/L; (NH_4_)_2_HPO_4_. VIII, extract content of 280 g/kg, inoculum content of 2.0 g/L; (NH_4_)_2_HPO_4_.

The effects of initial extract content in thick juice worts, the type of mineral supplement, and the dose of yeast inoculum on the dynamics and efficiency of alcoholic fermentation were determined. The obtained results are presented in Figures 1, 2, and 3.

In all the processing variants, the highest conversion of saccharose to fermentable sugars (expressed as reducing sugars) as a result of the activity of the β-fructofuranosidase (EC 3.2.1.26) present in yeast cells, was observed during the first 12 h of fermentation. Due to the prolonged initial phase of fermentation of worts with an extract content of 250 g/kg inoculated with a yeast dose of 1.0 g/L and supplemented with diammonium hydrogen phosphate (0.3 g/L of wort), ethanol production during the first 6 h of the process was very low (close to zero). In the next 6 h of the process, ethanol concentration increased to 0.8 ± 0.02% (v/v) (Figure 1A). Intensive biosynthesis of ethanol was reported after 12 h of fermentation. After 94 h of fermentation, ethanol concentration in the wort reached 12.4 ± 0.3% (v/v). On completion of the process, the real extract concentration of wort decreased from 250 g/kg to approximately 50 g/kg (by 80%). Also, the concentration of residual reducing sugars was still sufficiently high and amounted to 35.4 g/L of wort. This indicates that the sugar substrates were not fully utilized (Figure 1A).

Yeast cells have specific growth requirements leading to an imbalance or limitations resulting in incomplete fermentation. These requirements include specific levels of nitrogen, carbon, vitamins, water, oxygen, and metal ions. Metal ions are required for a number of purposes; they include bulk elements (such as magnesium, calcium, and potassium) and trace elements (such as zinc, copper, and manganese) needed by yeast cells [15]. Magnesium is necessary for the activation of several glycolytic enzymes, and in practical terms this means that if industrial media are magnesium-limited, the conversion of sugar to alcohol may be suppressed, leading to slow or incomplete fermentation processes [16].

Due to the incomplete fermentation of the wort with an initial extract content of 250 g/kg supplemented with only diammonium hydrogen phosphate and inoculated with 1 g yeast per L of wort, it seemed appropriate to conduct further fermentation experiments using mixed nutrients for yeast in the form of diammonium hydrogen phosphate (NH_4_)_2_HPO_4_ (0.3 g/L) and magnesium sulfate heptahydrate (MgSO_4_· 7 H_2_O) (0.1 g/L) and larger amounts of yeast inoculum.

Supplementation of thick juice wort (with an extract content of 250 g/kg) with MgSO_4_· 7 H_2_O (in addition to diammonium hydrogen phosphate) did not significantly improve the course of the process, so its efficiency was comparable to the process conducted in the presence of only diammonium hydrogen phosphate. Upon completion of this process, ethanol concentration in the wort supplemented with Mg^2+^ ions reached 13.0 ± 0.4% (v/v) and was not statistically higher than that in wort without the addition of MgSO_4_· 7 H_2_O (12.4 ± 0.2% v/v, 0.05 < *P* <0.10). Probably, the applied dose of magnesium salt (0.1 g MgSO_4_· 7 H_2_O contains 9.8 mg Mg^2+^) was too low to observe a beneficial effect of the metal ions on both the fermentation activity of yeast and the concentration of ethanol.

Rees and Stewart [15] proved that the addition of Mg^2+^ (500 ppm) to malt worts resulted in favorable changes to key fermentation parameters, leading to enhanced viability and increased cell numbers of yeast as well as to an initially increased rate of fermentation and ethanol production. However, it should be noted that an increase in ethanol production in the fermentation experiments conducted by Rees and Stewart [15] was not high (despite the differences being statistically significant). For example, oxygenated ale fermentation supplemented with magnesium chloride (Mg^2+^ concentration of 500 ppm) exhibited a total increase in ethanol production of 0.19% (v/v) at a lower gravity (12° Plato), and 0.31% (v/v) at a higher gravity (20° Plato). The fermentation of non-oxygenated wort in the presence of 500 ppm Mg^2+^ resulted in an increase in ethanol concentration (compared to the control sample) of 0.06% (v/v) at a lower wort gravity and 0.1% (v/v) at a higher wort gravity. Therefore, it is necessary to consider the composition of mineral nutrients for the yeast during alcoholic fermentation in order to avoid an unnecessary increase in production costs without attaining a substantial improvement in process efficiency.

A relationship between inoculum dose and ethanol concentration was observed for worts with an extract content of 250 g/L and 280 g/L. An increase in the pitching rate (inoculum dose) from 1.0 g, to 1.5 to 2.0 g/L in successive fermentation experiments, was reflected in a higher ethanol production and sugars consumption (Figure 1C-D). The final ethanol content in fermentation trials increased from 12.4 ± 0.2% (v/v) (inoculum dose of 0.1 g/L) to 13.3 ± 0.4% (v/v) in the wort inoculated with 1.5 g of yeast/L ( 0.02 < *P* <0.05) and to 14.2 ± 0.4% (v/v) in the wort with 2.0 g yeast/L (0.001 < *P* <0.01). Moreover, the content of residual sugars in the worts inoculated with larger amounts of yeast (1.5 and 2.0 g/L) ranged between 7.8 ± 0.2 and 17.2 ± 0.5 g/L and was relatively low in comparison to 35.4 ± 1.0 g/L of wort fermented with a pitching rate of 0.1 g/L (Figure 1).

The results of fermentation of worts with an extract content of 280 g/kg showed that their high gravity affected the course and results of fermentation. An increase in extract from 250 to 280 g/kg inhibited the fermentation activity of yeast and caused a gradual decrease in ethanol production. The lowest ethanol content (11.6 ± 0.3% v/v), its yield expressed as a percentage of the theoretical yield (72.3 ± 2.2%), and sugars intake (74.4 ± 2.2%) were found in a wort supplemented with diammonium hydrogen phosphate and inoculated with a yeast dose of 1.0 g/L (Figure 2A). As in the case of worts with a density of 250 g/kg, the addition of MgSO_4_· 7 H_2_O to worts with an extract content of 280 g/kg did not significantly improve process efficiency (0.05 < *P* <0.01). The highest sugars intake (88.9 ± 2.7%) and ethanol yield (86.0 ± 2.6% of the theoretical yield) were obtained in worts fermented by 2.0 g of yeast per 1.0 L of wort (Figure 2D, Figure 3).

A comparison of the fermentation results for all fermentation batches showed that the highest sugars intake (96.5 ± 2.9%) and ethanol yield (94.9 ± 2.8% of the theoretical yield) were observed in the wort with an extract content of 250 g/kg inoculated with 2 g of yeast per 1.0 L and supplemented with diammonium hydrogen phosphate (Figure 3). Despite the higher intake of sugars (91.4 ± 2.7%) in the sample supplemented with MgSO_4_· 7 H_2_O (0.1 g/L) than in the sample without the addition of Mg^2+^ ions (84.5 ± 2.5%), the yield of ethanol reached 86.8 ± 2.6% of the theoretical value and was not statistically higher than that obtained for the reference wort without the addition of Mg^2+^ (82.8 ± 2.5% of the theoretical yield, 0.10 < *P* <0.20).

The results obtained for all fermentation batches of 280 g/kg worts (with different yeast inoculum) were statistically significantly lower than those obtained for analogous fermentation trials with an extract content of 250 g/kg of wort. Based on two initial extract values used in our experiments and the fermentation results, the lower extract value, that is, 250 g/kg, was more favorable as high fermentation-activity of yeast was observed, enabling high utilization of fermentable sugars and maximal ethanol yield under the cited experimental conditions (Figure 3).

The obtained results are in accordance with the findings of Takeshige and Ouchi [17], who reported inhibited yeast growth and reduced ethanol yield in the process of molasses wort fermentation containing sugar at a concentration of 300 g/kg. Moreover, Dodić *et al.*[18], who fermented thick juice worts, observed that with an increase of fermentable sugars content from 5% to 20% (w/w), ethanol yields also increased for both investigated raw materials (molasses and thick juice). However, when an initial sugars content of 20% (w/w) was increased to 25% (w/w), the yields dropped significantly, from 67 to 56%. The low yields obtained by Dodić *et al.*[18] could have been caused by the fact that fermentation was carried out using baker’s yeast, which was most likely not adapted to high-density worts. Furthermore, the worts were not supplemented with mineral nutrients for yeasts.

Hinková and Bubník [19], who fermented concentrated raw sugar beet juice achieved the highest ethanol yield (88.2 to 94.4% of theoretical yield) when the sugar concentration in the wort amounted to 200 g/kg. The efficiency of fermentation and ethanol yield decreased with an increase in wort extract. The distillery yeast strains tested by Hinková and Bubník [19] showed an increased tolerance to osmotic pressure and provided higher yields in worts with higher initial concentrations of sugar. At high sugar concentrations, it was observed that the yeast experienced osmotic pressure, which led to plasmolysis and a lower ethanol yield [20]. Based on the obtained fermentation coefficients for the studied thick juice worts (Figures 1, 2, 3), the quantity of 100% (v/v) ethanol obtained from 100 kg of this raw material was calculated.

The results show that 38.9 ± 1.2 L 100% (v/v) ethyl alcohol could be produced from 100 kg of thick juice under the following favorable conditions, established in our experiments: extract content of 250 g/kg, yeast dose of 2 g/L of wort and (NH_4_)_2_HPO_4_ addition of 0.3 g/L of wort.

### Analysis of the chemical composition of the obtained distillates

The quality of bioethanol used for fuel purposes is strictly defined by the Polish national and industrial norms. High concentrations of fermentation by-products can cause a lower price of the final product. According to some producers of dehydrated ethanol, higher concentrations of pollutants in the raw spirit (unpurified ethyl alcohol) can cause fast deterioration of molecular sieves used in the process of ethanol dehydration [21]. The chemical composition of distillates obtained is shown in Table 2. Methanol concentration in the obtained raw spirits was low and ranged from 7.7 ± 0.7 to 9.3 ± 0.9 mg/L 100% (v/v) ethyl alcohol (no statistically significant differences, 0.05 < *P* <0.10).

**Table 2:** Chemical composition of distillates obtained from the fermentation of thick juice worts

**Compound (mg/L 100% v/v ethyl alcohol)**	**Parameters of fermentation**							
	**Extract content of 250 g/kg**				**Extract content of 280 g/kg**			
	**Inoculum content of 1.0 g/L; (NH**_**4**_**)**_**2**_**HPO**_**4**_	**Inoculum content of 1.0 g/L; (NH**_**4**_**)**_**2**_**HPO**_**4**_ **+ MgSO**_**4 **_**·7H**_**2**_**O**	**Inoculum content of 1.5 g/L; (NH**_**4**_**)**_**2**_**HPO**_**4**_	**Inoculum content of 2.0 g/L; (NH**_**4**_**)**_**2**_**HPO**_**4**_	**Inoculum content of 1.0 g/L; (NH**_**4**_**)**_**2**_**HPO**_**4**_	**Inoculum content of 1.0 g/L; (NH**_**4**_**)**_**2**_**HPO**_**4**_ **+ MgSO**_**4**_ **· 7H**_**2**_**O**	**Inoculum content of 1.5 g/L; (NH**_**4**_**)**_**2**_**HPO**_**4**_	**Inoculum content of 2.0 g/L; (NH**_**4**_**)**_**2**_**HPO**_**4**_
Methanol	8.1 ± 0.8^a^	7.7 ± 0.7^a^	9.3 ± 0.9^a^	7.7 ± 0.8^a^	9.3 ± 0.9^a^	8.1 ± 0.8^a^	9.3 ± 0.9^a^	9.1 ± 0.9^a^
Acetaldehyde	763.4 ± 4.2^a^	1626.0 ± 4.1^b^	2226.0 ± 6.5^c^	2214.5 ± 6.3^c^	3214.5 ± 7.5^d^	3869.8 ± 8.8^e^	3971.8 ± 9.2^f^	4172.9 ± 9.8^g^
Methyl acetate	8.2 ± 0.8^a^	9.2 ± 0.8^a^	8.6 ± 0.8^a^	9.3 ± 0.8^a^	7.9 ± 0.8^a^	9.0 ± 0.8^a^	8.3 ± 0.8^a^	9.2 ± 0.8^a^
Ethyl acetate	266.3 ± 2.5^b^	248.7 ± 2.5^a^	284.5 ± 2.8^d^	270.8 ± 2.6^c^	248.7 ± 2.5^a^	246.8 ± 2.5^a^	243.7 ± 2.5^a^	285.0 ± 2.8^d^
Isoamyl acetate	0.0^a^	0.0^a^	0.0^a^	1.9 ± 0.2^b^	2.6 ± 0.2^c^	4.2 ± 0.3^d^	7.1 ± 0.5^e^	8.7 ± 0.5^f^
Ethyl butyrate	33.5 ± 0.9^c^	45.3 ± 1.4^d^	60.1 ± 1.5^f^	52.1 ± 1.4^e^	18.0 ± 0.6^a^	18.6 ± 0.6^a^	21.9 ± 0.8^b^	22.6 ± 0.8^b^
n-propanol	155.9 ± 2.2^bc^	177.2 ± 2.8^e^	187.1 ± 2.8^f^	158.9 ± 1.6^c^	152.9 ± 1.6^ab^	150.3 ± 1.5^a^	206.1 ± 2.7^g^	165.9 ± 1.5^d^
2-methyl-1-propanol	314.3 ± 3.2^b^	307.2 ± 2.8^a^	368.9 ± 3.5^d^	381.1 ± 3.5^e^	357.8 ± 3.5^c^	402.2 ± 3.8^f^	355.7 ± 3.5^c^	355.3 ± 3.5^c^
n-butanol	7.4 ± 0.5^a^	7.8 ± 0.5^ab^	7.5 ± 0.5^a^	7.5 ± 0.5^a^	6.8 ± 0.5^a^	7.6 ± 0.5^a^	8.9 ± 0.7^b^	8.1 ± 0.6^b^
2-methyl-1-butanol	236.3 ± 2.5^a^	249.4 ± 2.5^b^	299.7 ± 2.9^e^	269.6 ± 2.7^c^	272.1 ± 2.7^c^	283.2 ± 2.7^d^	284.5 ± 2.7^d^	306.7 ± 3.2^f^
3-methyl-1-butanol	650.7 ± 3.5^a^	667.7 ± 3.8^b^	805.8 ± 4.1^d^	818.2 ± 4.2^e^	719.4 ± 3.8^c^	906.8 ± 4.2^g^	969.0 ± 4.3^h^	879.7 ± 4.1^f^

Results expressed as mean values ± standard error (n = 3). ^a-h^Mean values in lines with different letters are significantly different (*P* <0.05).

The aldehydes contained in spirits and alcoholic beverages are intermediates of two-step decarboxylation of alpha-keto acids to alcohols. The concentration of carbonyl compounds in raw spirits depends on the quality of raw materials, their chemical composition, and microbial contamination. Additionally, the final concentration of aldehydes and ketones is also affected by the technological processes. The parameters of wort fermentation, such as pH, temperature, and sugars concentration, affect the efficiency of enzymatic processes, including the conversion of glucose to pyruvate, its decarboxylation to acetaldehyde, and the reduction of the latter to ethanol [22]. The activity of the enzymes involved in these bioconversions can be decreased by a deficiency of certain microelements, for example, magnesium. This, in turn, can retard fermentation and lead to an accumulation of aldehydes in the fermented wort [23]. Acetaldehyde was the most abundant aliphatic carbonyl compound contained in the obtained raw spirits. Agricultural distillates derived from fermented thick juice worts with an extract content of 250 g/kg contained less acetaldehyde (763.4 ± 4.2 to 2226.0 ± 4.5 mg/L 100% v/v ethyl alcohol) than raw spirits obtained from worts with an extract content of 280 g/kg (3214.5 ± 7.5 to 4172.9 ± 9.8 mg/L 100% v/v ethyl alcohol, *P* <0.001). The addition of magnesium ions (in the form of MgSO_4_· 7 H_2_O ) to the studied thick juice worts had no beneficial effect and did not result in reduction of acetaldehyde synthesis. Moreover, greater amounts of yeast inoculum caused an increase in the synthesis of acetaldehyde. Our results concerning the content of this aldehyde are in line with the ones described by Gumienna *et al.*[4], who also studied the effects of extract concentration in thick juice worts on the course of fermentation and chemical composition of raw spirit (unpurified ethyl alcohol). The results of their investigation proved that an increase in sugar concentration in the fermentation medium increased the content of acetaldehyde. Most likely, an elevated osmotic pressure in the fermentation medium inhibits the activity of alcohol dehydrogenase (EC 1.1.1.1), which catalyzes the reduction of acetaldehyde to ethanol during alcoholic fermentation.

Ethyl acetate was the most abundant among the esters quantified in the distillates; its concentrations ranged from 243.7 ± 2.5 to 285.0 ± 2.8 mg/L 100% (v/v) ethyl alcohol. Also, low amounts of methyl acetate (7.9 ± 0.8 to 9.3 ± 0.8 mg/L 100% v/v ethyl alcohol), isoamyl acetate (0.0 to 8.7 ± 0.5 mg/L 100% v/v ethyl alcohol) and ethyl butyrate (18.0 ± 0.6 to 60.1 ± 1.5 mg/L 100% v/v ethyl alcohol) were found in the tested distillates.

All the distillates were enriched with higher alcohols, irrespective of the fermentation variant. Concentrations of n-propanol in the obtained raw spirits were statistically diverse and ranged from 150.3 ± 1.5 to 206.1 ± 2.7 mg/L 100% (v/v) ethyl alcohol while the content of 2-methyl-1-propanol was higher and ranged from 307.2 ± 2.8 to 402.2 ± 3.8 mg/L 100% (v/v) ethyl alcohol. The amounts of n-butanol in all the tested distillates were relatively small (6.8 ± 0.5 to 8.9 ± 0.7 mg/L 100% v/v ethyl alcohol). The most abundant isoamyl alcohol detected in the distillates was 3-methyl-1-butanol (650.7 ± 3.5 to 969.0 ± 4.3 mg/L 100% v/v ethyl alcohol), whereas the content of 2-methyl-1-butanol ranged from 236.3 ± 2.5 to 306.7 ± 3.2 mg/L 100% (v/v) ethyl alcohol (Table 2). Apart from the significantly higher levels of acetaldehyde in the distillates derived from worts with a density of 280 g/kg, there was no correlation between the concentrations of the identified byproducts and the fermentation conditions (Table 2).

The literature provides scant reports on the chemical composition of raw spirits originating from the intermediate products of sugar beet processing. Raw spirits obtained from the fermentation of thick juice worts were characterized by a lower content of higher alcohols than those obtained by Balcerek and Pielech-Przybylska [11] following the fermentation of starch mashes (from triticale) with the Ethanol Red^®^ yeast strain.

## Conclusions

The results of our study prove that the intermediate products of sugar beet processing, such as thick juice, may be considered an attractive raw material for bioethanol production. Saccharose is the principal component of its extract, so the only necessary operations before alcoholic fermentation are dilution, pH regulation, and addition of mineral nitrogen sources (if needed). The fermentation of thick juice worts with an extract content of 250 g/kg using 2 g of the dry distillery yeast Ethanol Red^®^ (*S. cerevisiae)* per 1 L of wort supplemented with (NH_4_)_2_HPO_4_ as a nutrient for yeast was determined to be favorable, as it enabled a high ethanol yield (38.9 ± 1.2 L 100% v/v ethyl alcohol from 100 kg of thick juice).

Due to limitations on sugar manufacturing in EU countries, the capacity of sugar factories is not fully utilized and they are ready to increase the processing of sugar beet into intermediates, which could serve as feedstock for bioethanol factories. This would be an alternative to starch processing, especially in the years of crop failures. Another crucial issue is also the ability of biofuels to reduce greenhouse gas (GHG) emissions. GHG emissions in the life cycle of bioethanol depend, among others, on the raw material and technology of production. The production of ethanol from sugar beet intermediate products is very favorable in that it lowers GHG emissions. The results of the study presented in this manuscript are aimed to improve the production process leading to measurable effects in terms of higher reduction of GHG emissions.

## Methods

### Raw material and microorganisms

Thick sugar beet juice was obtained from Dobrzelin Sugar Factory (Dobrzelin, Poland). Fermentation was carried out using a preparation of Ethanol Red^®^ dry distillery yeast (*S. cerevisiae)*, (Fermentis Division S.I.) designed for the production of alcohol up to 18% (v/v) at high temperature (35°C). The number of living cells at packing was >2.0 × 10^10^ per g, as declared by the manufacturer.

### Preparation of fermentation worts

Fermentation worts were prepared by diluting thick juice with distilled water, initially at a ratio of 1:1 w/w, and then obtaining solutions with an extract content of either 250 or 280 g/kg. The worts were acidified with 25% (w/w) sulfuric acid to pH 4.8 and supplemented with (NH_4_)_2_HPO_4_ (0.3 g/L) only or with (NH_4_)_2_HPO_4_ (0.3 g/L) and MgSO_4_ · 7 H_2_O (0.1 g/L) as nutrients for yeast.

### Fermentation variants

The fermentation variants were as follows:

I. Extract content of 250 g/kg; inoculum content of 1.0 g/L; (NH_4_)_2_HPO_4_

II. Extract content of 250 g/kg; inoculum content of 1.0 g/L; (NH_4_)_2_HPO_4_ + MgSO_4_ · 7 H_2_O

III. Extract content of 250 g/kg; inoculum content of 1.5 g/L; (NH_4_)_2_HPO_4_

IV. Extract content of 250 g/kg; inoculum content of 2.0 g/L; (NH_4_)_2_HPO_4_

V. Extract content of 280 g/kg; inoculum content of 1.0 g/L; (NH_4_)_2_HPO_4_

VI. Extract content of 280 g/kg; inoculum content of 1.0 g/L; (NH_4_)_2_HPO_4_ + MgSO_4_ · 7 H_2_O

VII. Extract content of 280 g/kg; inoculum content of 1.5 g/L; (NH_4_)_2_HPO_4_

VIII. Extract content of 280 g/kg, inoculum content of 2.0 g/L; (NH_4_)_2_HPO_4_

Fermentation experiments were carried out in 6-L glass flasks, each containing approximately 3 L of wort. After inoculation with yeast, which was preliminarily rehydrated, the flasks were closed with stoppers equipped with fermentation pipes filled with glycerol and kept in a thermostat-controlled room at 35°C. The process was carried out over 4 days (96 h). During the fermentation, samples for analysis were collected and the concentration of ethanol, real extract (after ethanol distillation), reducing sugars, and saccharose was measured, allowing us to compare the dynamics and biotechnological factors of the entire process.

### Distillation

When fermentation was complete, all ethanol was distilled from worts using a laboratory distillation unit consisting of a distillation flask, a Liebig cooler, a flask for collecting ethanol, and a thermometer. Raw spirits containing 20 to 23% (v/v) ethanol were refined to approximately 43% (v/v) in distillation apparatus equipped with a bi-rectifier unit (dephlegmator according to Golodetz), and subjected to chemical analysis.

### Analytical methods

Thick juice was analyzed by the methods recommended for the sugar industry [24]. Solid substance (total extract) was measured by using a hydrometer, which indicates the concentration of dissolved solids, mostly sugars, calibrated in g of saccharose per kg of water solution. Total nitrogen was determined by the Kjeldahl method. Volatile acids (expressed as acetic acid) were assayed using steam distillation. Reducing sugars and total sugars (after inversion with hydrochloric acid) were estimated by the Lane-Eynon method. Both were expressed in g of invert sugar per kg of thick juice. Saccharose concentration was calculated as the difference between total sugars and reducing sugars (taking into consideration a conversion coefficient of 0.95). Also pH was measured (with a digital pH-meter).

Worts were analyzed before and after fermentation using methods recommended for distilleries. Prior to fermentation, the worts were analyzed for pH, total extract, and reducing sugars (expressed as invert sugar) and saccharose content. On completion of fermentation, the worts were analyzed for real extract (after ethanol distillation), ethanol concentration in wort (using a hydrometer with a scale in % v/v of ethanol) and sugars content.

Distillates were analyzed using the Agillent 6890 N gas chromatograph (USA, Wilmington) equipped with a flame-ionization detector (FID), a split/splitless injector and an HP-Innowax capillary column (60 m × 32 mm × 0.5 μm). The temperature at the injector (split 1:45) and FID was kept at 250°C. The temperature program was as follows: 40°C (6 minutes), an increase to 83°C (2°C/minutes) and then to 190°C (5°C/minutes) (2 minutes). The flow rate of the carrier gas (helium) through the column was 2 mL/minute.

### Fermentation evaluation

The intake of total sugars (the percentage yield of sugar consumption during fermentation) was calculated as a ratio of sugars used during the fermentation to their content in the wort prior to this process, and expressed in percent. The yield of ethanol was calculated according to the stoichiometric Gay-Lussac equation in relation to total sugars and expressed as a percentage of the theoretical yield.

### Statistical analysis

All samples were prepared and analyzed in triplicate. Statistical analysis was carried out using the Micromal Origin ver. 6.0 software (Northampton, USA).

## Abbreviations

(NH4)2HPO4: Diammonium hydrogen phosphate; MgSO4 · 7 H2O: Magnesium sulfate heptahydrate; FID: Flame-ionization detector; GHG: Greenhouse gas; VHG: Very high gravity.

## Competing interests

The authors declare that they have no competing interests.

## Authors’ contributions

PD and MB designed the experiments. MB, KP-P and PP performed the experiments. MB and KP-P wrote the paper. PD and PP were involved in the evaluation of results and review of the paper. All authors read and approved the final manuscript.

## References

[B1] Liew FM Köpke M Simpson SD Fang Z Gas fermentation for commercial biofuels production. Chapter 5 Liquid, gaseous and solid biofuels-conversion techniques DOI: 10.5772/52164. [http://www.intechopen.com/books/liquid-gaseous-and-solid-biofuels-conversion-techniques/gas-fermentation-for-commercial-biofuels-production. Publisher: InTech, Chapters published March 20, 2013 under CC BY 3.0 license

[B2] Lin Y Tanaka S Ethanol fermentation from biomass resources: current state and prospects Appl Microbiol Biotechnol 2006 69 627 642 10.1007/s00253-005-0229-x 16331454

[B3] Szopa J Patelski P Biotechnological trends in sugar beet processing [article in Polish] Sug Ind Mag 2006 11 326 327

[B4] Gumienna M Lasik M Czarnecki Z Szambelan K Applicability of unconventional energy raw materials in ethanol productions Acta Sci Pol Technol Aliment 2009 8 18 24

[B5] Leiper KA Schlee C Tebble I Stewart GG The fermentation of beet sugar syrup to produce bioethanol J Inst Brew 2006 112 122 133 10.1002/j.2050-0416.2006.tb00242.x

[B6] Zhao XQ Bai FW Mechanisms of yeast stress tolerance and its manipulation for efficient fuel ethanol production J Biotechnol 2009 144 23 30 10.1016/j.jbiotec.2009.05.001 19446584

[B7] Piddocke MP Kreisz S Heldt-Hansen HP Nielsen KF Olsson L Physiological characterization of brewer’s yeast in high-gravity beer fermentations with glucose or maltose syrups as adjuncts Appl Microbiol Biotechnol 2009 84 453 464 10.1007/s00253-009-1930-y 19343343

[B8] Bai FW Anderson WA Moo-Young M Ethanol fermentation technologies from sugar and starch feedstocks Biotechnol Adv 2008 26 89 105 10.1016/j.biotechadv.2007.09.002 17964107

[B9] Mussatto SI Dragone G Guimarães PMR Silva JP Carneiro M Roberto IC Vicente A Domingues L Teixeira JA Technological trends, global market, and challenges of bio-ethanol production Biotechnol Adv 2010 28 817 830 20630488 10.1016/j.biotechadv.2010.07.001

[B10] Balcerek M Pielech-Przybylska K Patelski P Selection of yeast strains for alcoholic fermentation of sugar beet thick juice and greek syrup Biom Bioenerg 2011 12 4751 4910

[B11] Balcerek M Pielech-Przybylska K Effect of simultaneous saccharification and fermentation conditions of native triticale starch on the dynamics and efficiency of process and composition of the distillates obtained J Chem Technol Biotechnol 2013 4 615 622

[B12] Fermentis Ethanol Red^®^ Dry alcohol yeast http://www.fermentis.com/wp-content/uploads/2012/06/EthanolRED_EN.pdf

[B13] Ranković J Dodić J Dodić S Popov S Bioethanol production from intermediate products of sugar beet processing with different types of Saccharomyces cerevisiae Chem Ind & Chem Eng Quarterly 2009 15 13 16 10.2298/CICEQ0901013R

[B14] Hoffmann C Marlander B Components of harmful nitrogen in sugar beet-influence of variety and environment Proceedings of the 1st joint 1/RB-ASSBT Congress 2003 San Antonio (USA): Session Physiology and Biotechnology 429 434

[B15] Rees EMR Stewart GG Effects of magnesium, calcium and wort oxygenation on the fermentative performance of ale and lager strains fermenting normal and high gravity worts J Inst Brew 1999 105 211 217 10.1002/j.2050-0416.1999.tb00021.x

[B16] Walker GM The roles of magnesium in biotechnology Crit Rev Biotechnol 1994 14 311 354 10.3109/07388559409063643 7889576

[B17] Takeshige K Ouchi K Effects of yeast invertase on ethanol production in molasses J Ferm Bioeng 1995 79 513 515 10.1016/0922-338X(95)91274-9

[B18] Dodić S Popov S Dodić J Ranković J Zavargo Z Mučibabić RJ Bioethanol production from thick juice as intermediate of sugar beet processing Biom Bioenerg 2009 33 822 827 10.1016/j.biombioe.2009.01.002

[B19] Hinková A Bubník Z Sugar beet as a raw material for bioethanol production Czech J Food Sci 2001 19 224 234

[B20] Marx S Brandling J van der Gryp P Ethanol production from tropical sugar beet juice Afr J Biotechnol 2012 54 11709 11720

[B21] Kłosowski G Mikulski D Czupryński B Kotarska K Characterisation of fermentation of high-gravity maize mashes with the application of pullulanase, proteolytic enzymes and enzymes degrading non-starch polysaccharides J Biosci Bioeng 2010 109 466 471 10.1016/j.jbiosc.2009.10.024 20347769

[B22] Kłosowski G Czupryński B Reasons of formation of carbonyl compounds with particular consideration of acetaldehyde [article in Polish] Przem Ferm Owoc-Warz 1993 37 8 10

[B23] Łączyński B Reasons of too high concentration of aldehydes in raw spirits and methods of prevention in agricultural distilleries [article in Polish] Przem Ferm Owoc-Warz 1995 39 13 14

[B24] AOAC Official Methods of Analysis of AOAC International Volume 16 1995 2 Maryland USA: AOAC International Methods: 906.03; 920.176; 930.36; 932.14; 964.08; 968.28

